# Utility of Serum 1,3-β-d-Glucan Testing for Diagnosis and Prognostication in COVID-19-Associated Pulmonary Aspergillosis

**DOI:** 10.1128/spectrum.01373-22

**Published:** 2022-05-31

**Authors:** Matthias Egger, Florian Prüller, Robert Krause, Juergen Prattes, Martin Hoenigl

**Affiliations:** a Division of Infectious Diseases, Department of Internal Medicine, Medical University of Graz, Graz, Austria; b BioTechMed-Graz, Graz, Austria; c Clinical Institute of Medical and Chemical Laboratory Diagnostics, Medical University of Graz, Graz, Austria; d Division of Infectious Diseases and Global Public Health, University of California San Diego, San Diego, California, USA; e Clinical and Translational Fungal-Working Group, University of California San Diego, San Diego, California, USA; f Department I of Internal Medicine, University Hospital of Cologne, Cologne, Germany; Universidade de Sao Paulo

**Keywords:** Covid-19, Covid-19-associated pulmonary aspergillosis, β-d-glucan, ICU, 3-beta-d-glucan, diagnostics

## LETTER

COVID-19-associated pulmonary aspergillosis (CAPA) has emerged as a life-threatening complication in patients admitted to intensive care units (ICUs) for COVID-19-associated acute respiratory failure (ARF). Two recent studies on the prognostic potential of serum 1,3-β-d-glucan (BDG) in ICU patients with COVID-19 ARF (1, 2) found positive serum BDG to be associated with 75% to 90% mortality, versus 42% to 47% mortality in those with negative serum BDG (*P* < 0.01; adjusted odds ratio of 1.3 per 10-point increase of BDG among patient with CAPA). While in some studies serum BDG results were also utilized as mycological evidence (3), the diagnostic potential of serum BDG for CAPA in ICUs remains controversial and larger analyses are lacking.

We conducted a retrospective single center study, analyzing 116 serum samples obtained from 69 consecutive ICU patients admitted with COVID-19 ARF at the University Hospital of Graz, Austria, between March 2020 and April 2021 for BDG (4). BDG was tested according to previously described methods using reagents from the Fungitell assay (Associates of Cape Cod, Falmouth, MA) (5). CAPA cases were classified according to 2020 ECMM/ISHAM consensus criteria (6). Statistical analyses were performed using SPSS 25 (SPSS Inc., Chicago, IL, USA). Sensitivity and specificity for CAPA versus no CAPA were calculated for the manufacturer-recommended BDG cutoff (positive if ≥80 pg/mL). For BDG, receiver operating characteristic (ROC) curve analyses were performed and area under the curve (AUC) values were calculated including 95% confidence intervals (CI) for the outcome mortality in the ICU. Fisher’s exact test was used to compare mortality in those with positive and those with negative BDG test results. A two-sided *P* value of <0.05 was taken as cutoff for statistical significance.

Three patients met criteria for probable CAPA, while 66 patients were classified as not having CAPA. Per-patient sensitivity and specificity of serum BDG for CAPA diagnosis are shown in Table 1. BDG positivity at ICU admission did not predict death in the ICU (AUC, 0.577; 95% CI, 0.44 to 0.71; 60% [6/10] mortality in those with positive BDG versus 42% [25/59] in those with negative BDG [*P* = 0.33]). For patients with two or more samples obtained, a single positive BDG test at any time point was associated with 75% mortality (6/8) versus 45% (10/22) in those with consistently negative BDG test results (*P* = 0.23).

**TABLE 1:** Sensitivity and specificity of serum BDG per patient (i.e., in patients with multiple samples a single positive serum BDG result was sufficient for classification as “BDG positive”), comparing proven/probable/possible CAPA versus no CAPA in ICU patients

Cohort	Proven/probable/possible CAPA vs no CAPA (% [no. positive/total no.])	
	Sensitivity	Specificity
Egger et al.^*a*^	0 (0/3)	85 (56/66)
Dellière et al. (1)	44 (20/45)	NA^*b*^
Ergün et al. (2)	74 (29/39)	42 (56/133)
Overall sensitivity/specificity	56 (49/87)	56 (112/199)

a Data presented within this work.

b NA, not available.

CAPA is characterized by tissue invasive growth in the lungs during early infection. Angioinvasion typically occurs only in later stages of the disease, resulting in limited sensitivity of serum biomarker testing (7). Combining results of our study with those from prior studies (1, 2), per-patient sensitivity and specificity of serum BDG for CAPA were 56% (49/87) and 56% (112/199), respectively. While prevalence of CAPA varies, a median prevalence of 10% (4) and 15% (8) has been reported in the largest multicenter studies conducted to date. When applying calculated sensitivity/specificity to a prevalence of 10% or 15%, the positive predictive values (PPVs) of BDG for CAPA diagnosis are 12% and 18%, respectively, while negative predictive values (NPVs) are 92% and 88%. The PPVs and NPVs were therefore only marginally higher than the disease frequency itself, rendering the diagnostic performance equally effective as picking by chance.

While taking into account the relatively small sample size number, we conclude that serum BDG probably has no role in either diagnosing or ruling out CAPA in settings with prevalence rates below 15%. Larger studies are needed to confirm that finding. While BDG measured at the time of ICU admission was also lacking prognostic potential, BDG may, to some extent, predict survival in COVID-19 ICU patients, particularly when measured closer to the fatal event, in its function as a marker of the leaky gut (9, 10).
